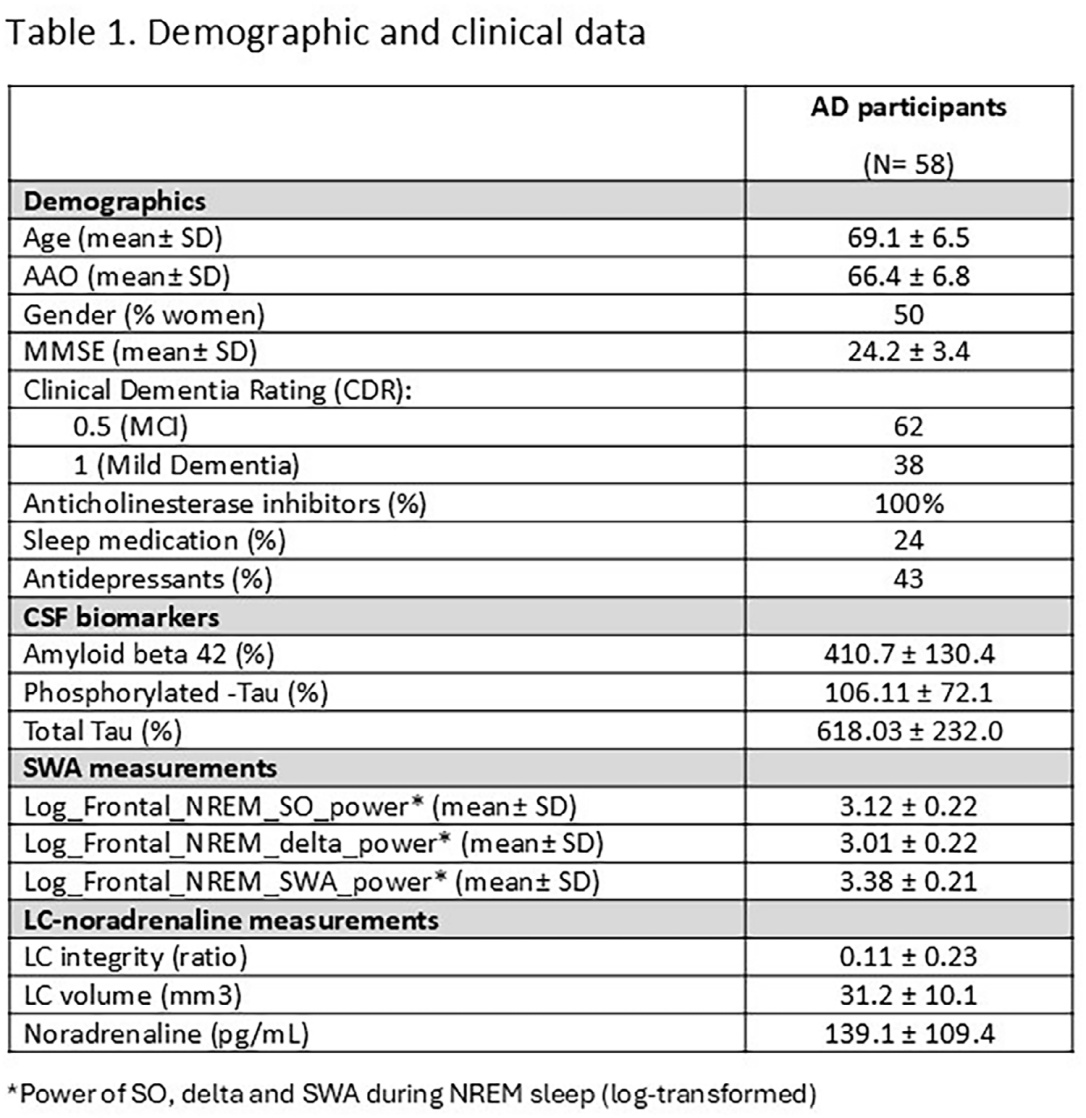# Role of Locus Coeruleus Integrity in Slow‐Wave Activity in Alzheimer's Disease

**DOI:** 10.1002/alz70857_096973

**Published:** 2025-12-24

**Authors:** Neus Falgàs, Núria Tort‐Colet, Andrea Val‐Guardiola, Marta Peña, Gerard Mayà, Carles Gaig, Beatriz Bosch‐Capdevila, Aurora Arqueros, Agnès Pérez‐Millan, Gerard Piñol‐Ripoll, Albert Lladó, Manuel Morales‐Ruiz, Emma Muñoz‐Moreno, Lea T. Grinberg, Oriol Grau‐Rivera, Álex Iranzo, Raquel Sánchez‐Valle

**Affiliations:** ^1^ Unitat d'Alzheimer i Altres Trastorns Cognitius, Servei de Neurologia, Hospital Clínic, Barcelona, Spain; ^2^ Global Brain Health Institute, San Francisco, CA, USA; ^3^ Barcelonaβeta Brain Research Center (BBRC), Pasqual Maragall Foundation, Barcelona, Spain; ^4^ Alzheimer's disease and other cognitive disorders Unit. Hospital Clínic de Barcelona; FRCB‐IDIBAPS; University of Barcelona, Barcelona, Spain; ^5^ IDIBAPS, Barcelona, Barcelona, Spain; ^6^ Hospital Clínic, Barcelona, Spain; ^7^ Hospital Clínic de Barcelona, Barcelona, Spain; ^8^ Alzheimer's disease and other cognitive disorders Group. Service of Neurology, Hospital Clínic de Barcelona. Fundació Recerca Clínic Barcelona‐IDIBAPS, Barcelona, Spain; ^9^ Neurology Service, Hospital Clínic de Barcelona and Institut D'Investigacions Biomèdiques August Pi i Sunyer (IDIBAPS), Barcelona, Spain; ^10^ Alzheimer's disease and other cognitive disorders unit, Hospital Clínic, IDIBAPS, Barcelona, Spain; ^11^ Image Diagnostic Centre, IDIBAPS, Hospital Clínic de Barcelona, Barcelona, Spain, Barcelona, Spain; ^12^ Memory and Aging Center, UCSF Weill Institute for Neurosciences, University of California, San Francisco, San Francisco, CA, USA; ^13^ Alzheimer's Disease and Other Cognitive Disorders Unit, Hospital Clínic, Institut d’Investigacions Biomediques August Pi i Sunyer (IDIBAPS), Barcelona, Spain

## Abstract

**Background:**

Locus coeruleus (LC) degeneration, a hallmark of Alzheimer's disease (AD), occurs early in the disease course, preceding cognitive deficits. This noradrenergic nucleus of the brainstem is linked to tau pathology accumulation within key nuclei regulating sleep and behavior, contributing to prodromal neuropsychiatric symptoms such as depression and sleep disturbances. Slow‐wave activity (SWA) plays a crucial role in memory processing, both of which are impaired in AD. However, the relationship between LC degeneration and changes in slow‐wave sleep remains unclear.

**Method:**

We recruited 58 participants diagnosed with AD based on cerebrospinal fluid (CSF) biomarkers (*n* = 54) or amyloid‐PET (*n* = 4) at the Hospital Clínic de Barcelona. Participants underwent overnight video‐polysomnography (PSG) and neuromelanin‐sensitive MRI to assess LC integrity. CSF noradrenaline was measured using high‐performance liquid chromatography. Sleep parameters, including power in the slow oscillation (SO, 0.5–1 Hz), delta (1–4 Hz), and SWA (0.5–4 Hz) frequency bands, were computed from deep non‐rapid eye movement (NREM) sleep stages (N2 and N3) using PSG frontal electrodes (F3 and F4). Linear regression models evaluated the relationship between LC integrity or CSF noradrenaline and SWA, adjusting for age, sex, Clinical Dementia Rating (CDR), and prescribed medications.

**Result:**

The sample had a mean age of 69 years, balanced by sex (50% women), and included participants in early AD stages (mild cognitive impairment or mild dementia) (Table 1). Twenty‐four percent and 43% of participants were prescribed sleep and antidepressant medications, respectively. LC integrity was positively associated with SWA (β=0.27, *p* = 0.043), independent of age, sex, CDR, and medications. Similar trends were observed for SO (β=0.27, *p* = 0.059) and delta power (β=0.25, *p* = 0.07). No significant association was found between SWA and CSF noradrenaline levels.

**Conclusion:**

These findings suggest that LC integrity contributes to SWA alterations in AD, highlighting its potential role in the disruption of sleep‐related neurophysiological processes. This study emphasizes the importance of LC degeneration in the sleep pathology of AD and supports further exploration of LC‐targeted interventions to address sleep and cognitive deficits.